# Lifestyle and Ice: The Relationship between Ecological Specialization and Response to Pleistocene Climate Change

**DOI:** 10.1371/journal.pone.0138766

**Published:** 2015-11-04

**Authors:** Eva Kašparová, Anton P. Van de Putte, Craig Marshall, Karel Janko

**Affiliations:** 1 Laboratory of Fish Genetics, Institute of Animal Physiology and Genetics, Academy of Sciences of the Czech Republic, Rumburska 89, 27721 Libechov, Czech Republic; 2 Institute of Vertebrate Biology, Academy of Sciences of the Czech Republic, Kvetna 8, 603 65, Brno, Czech Republic; 3 Laboratory of Biodiversity and Evolutionary Genomics, Katholieke Universiteit Leuven, Ch. Deberiotstraat 32, B-3000 Leuven, Belgium; 4 Department of Biochemistry, and Genetics Otago, University of Otago, P.O. Box 56, Dunedin, New Zealand; 5 Centre for Polar Ecology, University of South Bohemia in Ceské Budejovice, Na Zlate stoce 3, 370 05, Ceske Budejovice, Czech Republic; 6 Life Science Research Centre, Department of Biology and Ecology, Faculty of Natural Sciences, University of Ostrava, Chittussiho 10, 710 00 Silesian Ostrava, Czech Republic; University of Padova, ITALY

## Abstract

Major climatic changes in the Pleistocene had significant effects on marine organisms and the environments in which they lived. The presence of divergent patterns of demographic history even among phylogenetically closely-related species sharing climatic changes raises questions as to the respective influence of species-specific traits on population structure. In this work we tested whether the lifestyle of Antarctic notothenioid benthic and pelagic fish species from the Southern Ocean influenced the concerted population response to Pleistocene climatic fluctuations. This was done by a comparative analysis of sequence variation at the cyt *b* and S7 loci in nine newly sequenced and four re-analysed species. We found that all species underwent more or less intensive changes in population size but we also found consistent differences between demographic histories of pelagic and benthic species. Contemporary pelagic populations are significantly more genetically diverse and bear traces of older demographic expansions than less diverse benthic species that show evidence of more recent population expansions. Our findings suggest that the lifestyles of different species have strong influences on their responses to the same environmental events. Our data, in conjunction with previous studies showing a constant diversification tempo of these species during the Pleistocene, support the hypothesis that Pleistocene glaciations had a smaller effect on pelagic species than on benthic species whose survival may have relied upon ephemeral refugia in shallow shelf waters. These findings suggest that the interaction between lifestyle and environmental changes should be considered in genetic analyses.

## Introduction

Climate change has always been a feature of the natural world. Although we can trace some of the physical effects of climate change, identifying the biological effects of past climatic change is complicated. Studies identifying ‘genetic wakes’[1] carrying signals of dispersal, extinction, and speciation correlating with Pleistocene glacial cycles have provided evidence of dramatic worldwide effects on species. However, no simple pattern of population response to these climatic changes has been found. Although explanations for different responses among species to Pleistocene climate change have been proposed in comparative studies [2,3] general patterns of response are not always present. Even closely-related organisms exposed to common events may have quite distinct population histories with no immediately obvious reasons for the differences [4]. Polar regions provide attractive natural laboratories to address such issues since polar ecosystems are relatively simpler than low-latitude ones and the direct effects of climatic changes are more easy to detect compared to lower latitudes (e.g. physical eradication of habitats by expanding glaciers). Despite ongoing research, our knowledge of evolution at the poles remains limited [5]. Moreover, studies tend to focus on the more easily accessible Northern Hemisphere than on the Southern. This is especially important since diversity patterns are not symmetrical between the two hemispheres [6] with much higher levels of endemism within the Southern polar circle, suggesting that different mechanisms apply. In the Arctic, despite evidence of high-latitude refuges in some organisms (e.g. cloudberry (*Rubus chamaemorus*) [7] both marine and terrestrial organisms were apparently able to move along a latitudinal cline in response to changes in the Pleistocene climate (2,5 Mya to 12 Kya) (e.g. relict moss *Rhytidium rugosum* [8] or molluscs [9]). In the Antarctic and the Southern Ocean, the situation is quite different. Populations are relatively isolated from other parts of the globe by the Antarctic Polar Front (APF) and by physiological and competitive constraints on potential invaders [5,9,10]. To understand the effects of future climate change it is useful to study how Antarctic organisms survived in isolation and coped with climate fluctuations and oscillating ice sheet cover during the Pleistocene.

Contemporary population genetic studies reveal no simple pattern of population history in the species on the Antarctic continental shelf and Southern Ocean. Some species demonstrate population structure implying historical fragmentation into isolated glacial refuges in the high-Antarctic that led either to partial reproductive isolation (e.g. crinoids, icefish, octopods [5,10–14] and secondary hybridization during interglacial periods (e.g. in notothenioids [15]), or to allopatric speciation (e.g. sea slug [16]). Other species show a ‘classical’ pattern suggesting survival of inhospitable periods in lower-latitude refugia and subsequent recolonisation of Antarctica from the north (e.g. bull kelp [17]). Some species show recent expansions associated with glacial retreat after the Last Glacial Maximum (LGM) (15–12 Kya) [18] (e. g. the limpet *Nacella* and trematomid fish [19,20] whereas shrimps, sea spiders and deep-sea echinoids demonstrate much older expansion timings [21–23]. Strikingly different population dynamics have been detected even among closely-related species e.g. within the Trematominae [20] and among springtails (Collembola) [24].

Similar discrepancies were found in studies that address interspecific processes. It has been suggested that ice ages might have caused the extinction of some species [25], or conversely, increased the speciation rate during the Pleistocene (glaciation as a diversity pump; [16]). On the other hand, a recent macroevolutionary study of the diversification rate among the fish tribe Trematominae demonstrated it was largely unchanged during the Pleistocene [26].

There may be several reasons for this diversity of patterns. Firstly, the genetic markers and analytical tools used in different studies may not be equivalent, and difficulties in calibrating the molecular clock of Antarctic organisms may hamper clear-cut comparisons among unrelated taxa [27] regardless of any underlying patterns. Alternatively, stochastic variability induced by asynchronous patterns of ice expansion and retreat in glacial periods may account for the variety of patterns of population structure when geographically isolated refugia on the continental shelf repeatedly appeared and disappeared. Differences in the timing of these may have allowed species to migrate among refugia [28]. In addition, the response of a species to glaciations may depend upon its distribution range and the local conditions within each of the Antarctic biogeographic provinces [24,29,30]. In contrast, several studies show that the ability of a species to cope with climatic changes mainly depends on ecological factors such as niche width, habitat preference, and depth range [20,23].

In order to understand the processes that lead to diverse population genetic patterns among marine species, Hellberg [4] asked: “Do closely related species differ in inferred effective population size? What are life histories or ecological attributes of this variation?” In this study we have addressed both of Hellberg’s questions. Specifically focusing on thirteen closely related but ecologically diverse fish species (Perciformes, Notothenioidei), we looked for different demographic responses to Pleistocene ice sheet expansions and tested whether the long-term effective population size and timing of population expansions has been governed by species-specific lifestyles such as the use of benthic or pelagic habitats.

The notothenioid fish are an excellent model for assessing the role of lifestyle in influencing demographic history. The origins of modern notothenioid fish date from Southern Ocean cooling during the Oligocene [31,32] when the Eocene fish fauna went extinct. This allowed the subsequent occupation of “vacant” niches by ancestral notothenioid fish that avoided freezing because of antifreeze glycoproteins (AFGP) (reviewed in Eastman & McCune [33]) [32]. These notothenioid fish radiated during the Miocene from a putative benthic ancestor without a swim bladder into an array of species strikingly diverse in morphology and lifestyle. A pelagic lifestyle seems to have arisen independently several times in the suborder [26,34] indicative of significant plasticity in body size and conformation. Notothenioid fish comprise closely related, monophyletic, and ecologically diverse species with a polyphyletic distribution of traits (including cryopelagic, pelagic and benthic lifestyles). These characteristics make them suited for comparative studies and minimize the effect of “phylogenetic constraints” sensu Gould and Lewontin [35] i.e. the risk that the differences among species result from intrinsic characteristics of particular phylogenetic lineages rather than the presence of some potentially adaptive trait.

This study extends previous results by re-analyzing the data from four species from Janko et al. [20] and by adding datasets from nine newly-sequenced species. Two genetic loci (mitochondrial cyt *b* and nuclear S7) were sequenced from all species and subjected to a number of analytical approaches to look for evidence of historical population changes. We sorted the thirteen fish species into distinct eco-groups according to their habitat specialization and predicted that if habitat use specialization affects species responses to climatic events, there should be evidence of consistent patterns in inferred demographic histories among similar eco-groups. Pelagic species are likely to be less affected by scouring of the shelf by glaciers and ice sheet advance, and may have even experienced population expansions during ice ages as the polar pelagic habitat expanded north with movement of the Antarctic Polar Front during the LGM [18,36]. By contrast, populations of benthic species are potentially more vulnerable to local extinctions caused by increasing glaciation and might have suffered more recent and severe bottlenecks associated with the last glaciation that led, for example, to reductions in the extent of shallow bottom.

## Results

### Genetic variability of species

Two markers, cytochrome *b* (cyt *b*) and nuclear S7 gene were PCR amplified and tested for selection effects. The S7 dataset was analysed as unrecombined regions including the gametic phases of heterozygous individuals.

The cyt *b* sequences showed no evidence of indels or stop codons. Recombination events were detected in several S7 datasets; these were subsequently pruned to obtain the longest non-recombined portion of that locus for each species (Table 1). In *Pagetopsis macropterus*, the high number of recombination events prevented phasing and obtaining sufficiently long sequences. Therefore, the S7 dataset of *Pagetopsis macropterus* was excluded from all analyses. No departures from neutral expectations for any species were found in the McDonald-Kreitman and HKA tests within cyt *b* and across both loci (Table 1).

**Table 1:** Results of neutrality tests and expansion rate estimation.

		Cyt *b*					Tests using both loci		S7 intron			
Species	E	N	L	Tajima´s D	Fu´s F_s_	McD&K	HKA	LAMARC	N	L	Tajima´s D	Fu´s F_s_
*Aethotaxis mitopteryx*	P	33	553	● -2.42273	● -14.642	0.175	5.51	795.41 +/- 294	46	571	●-2.36578	●-17. 377
*Notothenia rossii*	I	30	723	● -2.02488	● -9.332	0.763	0.0936	4325.29 +/-1324	26	188	-1.77006	●-7.83
*Trematomus eulepidotus*	I	35	399	● -2.29808	● -15. 249	0.763	5.63	610.8747 +/- 122	42	622	-1.7435	●-33.793
*Gobionotothen gibberifrons*	B	29	640	● -2.08520	● -12.746	0.002	0.268	35.09154 +/- 58	29	340	-1.82666	●-9.4
*Gymnodraco acuticeps*	B	23	732	● -1.97778	● -4.152	0.175	4.32	270.7582 +/- 236	42	273	-0.66043	-0.05
*Lepidonotothen nudifrons*	B	40	463	● -1.97088	● -8.233	1.586	0.1959	5682.365 +/- 1253	66	625	-0.07802	0.739
*Pagetopsis macropterus*	B	25	687	● -1.82397	● -4.992	0.763	NA	NA	NA	NA	NA	NA
*Trematomus hansoni*	B	76	399	-1.8338	● -8.681	3.643	4.43	403.455 +/- 240	98	479	-1.6701	●-16.978
*Trematomus nicolai*	B	34	353	● -1.72673	● -3.595	1.263	0.3768	49.74308 +/- 261	58	443	-1.04706	-0.465

Results here are for nine newly sequenced species. E—eco-group (P—pelagic, I—intermediate, B—benthic), N—number of sequenced species, L—length in base pairs of the non-recombined locus, McD&K—G value with Williams' correction of the McDonald & Kreitman test, HKA—sum of deviations of the HKA test, LAMARC—g with 95% support intervals with averaged errors, Symbol ● indicates values significant after sequential Bonferroni correction (α = 0,05), the *P*. *macropterus* S7 dataset was not available for this analysis.

Since geographical subdivisions can bias estimates of historical demography, AMOVA analyses were performed to check population connectivity between regions. Significant evidence of population structuration were found in *T*. *hansoni* cyt *b* and S7 datasets and in the *L*. *nudifrons* S7 dataset (see Table A in S1 File). For those species with evidence of geographical structure, we performed tests of genetic variability and neutrality tests according to the geographic subdivision for both markers (see Table B in S1 File). The detection of structure generally reflected a distinction between biogeographic provinces in *T*. *hansoni* (high-Antarctic vs Ant. Peninsula), whereas in *L*. *nudifrons* we noticed significant differences among individual sampling sites.

### Deviations from the constant population size model

**Fig 1:**
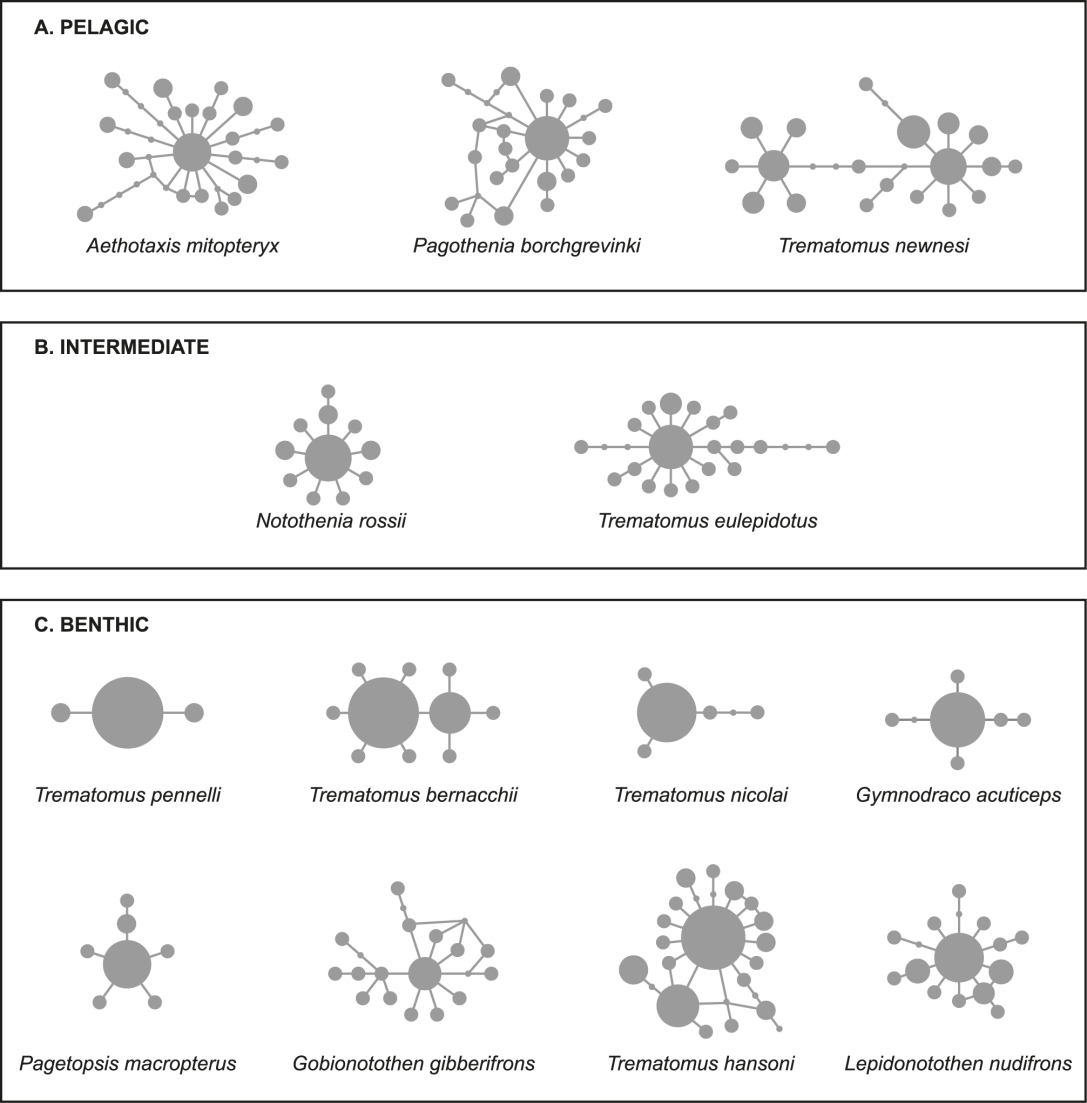
Unrooted networks of cyt *b* constructed by statistical parsimony. Unique haplotypes are represented by circles and their absolute frequency is indicated by circle size. Internodes represent unsampled inferred intermediate haplotypes.

A number of different methods were used to assess the deviation from a constant population size model. Significant deviations were observed in all cyt *b* datasets (Table 1) as suggested by significantly negative values of Fu’s F_s_ in all datasets and of Tajima’s D in all but one case (Table 1). This is consistent with the star-shaped haplotype networks (Fig 1) expected for species undergoing recent demographic expansion. Generally, we found that the haplotype networks of pelagic species are more complex than in benthic species. We did note however, that species with geographic structure (*T*. *hansoni*, *L*. *nudifrons* and also *G*. *gibberifrons*) possessed more complex patterns than other benthic species. Arlequin's goodness-of-fit statistics did not reject a sudden expansion model in any dataset suggesting that all species may have undergone an increase in population size. The estimated expansion time (in mutational units) differed for pelagic and benthic species. Estimates from DnaSP for pelagic species ranged from 1.22 to 2.23, and for benthic from 0.08 to 1.53 (see Table 2). Estimates from SITES matched those of DnaSP very closely except for *G*. *acuticeps* and *T*. *nicolai* where SITES estimates exceeded those of DnaSP almost seven-fold. On the other hand, although Arlequin estimates were similar to those of SITES and especially those of DnaSP in most species, they differed by as much as twenty-fold for *T*. *pennellii*, *T*. *nicolai* and *G*. *acuticeps* (see S1 Fig).

**Table 2:** Summary of genetic diversity estimates and of population expansion times for cyt b and S7 of a combined dataset of 13 species.

		**Cyt b**					
**Species**	**E**	**hd**	π *10 ^**3**^	ϴW*10 ^**3**^	T(ex) (DSP)/T(ex) (S) /T(ex) (A)*10 ^**3**^	**T(ex) (DSP)/T(ex) (S) /T(ex) (A) (Kya)**	**DS**
*Aethototaxis mitopteryx*	P	0.87	4.8	14.95	1.22/ 2.32/ 1.15 (0.18–4.39)	148.53/ 282.38/ 140.24 (21.95–535.37)	N
*Pagothenia borchgrevinki*	P	0.88	3.6	8.5	1.68/ 1.79/ 1.46 (0.98–2.06)	166.16/ 168.73/ 144.55 (97.03–203.96)	J
*Trematomus newnesi*	P	0.91	6.9	9.77	2.23/ 4.69/ 5.52 (0.86–9.67)	300.77/ 633.71/ 745.94 (116.22–1306.756)	J
*Notothenia rossii*	I	0.68	1.3	3.49	0.63/ 0.65/ 0.73(0.32–1.33)	62.93/ 65.17/ 73 (32–133)	N
*Trematomus eulepidotus*	I	0.86	7.6	21.35	1.47/ 4.4/ 2.51(0.00–7.7)	179.42/ 492.14/ 306.1 (0–939.022)	N
*Gobionotothen gibberifrons*	B	0.86	3.1	7.22	1.53/ 2.21/ 1.63 (0.00–3.71)	160.61/ 232.2/ 171.58 (0–390.52)	N
*Gymnodraco acuticeps*	B	0.32	0.7	2.12	0.08/ 0.55/ 2.05 (0.25–2.82)	9.99/ 67.26/ 250 (30.49–343.90)	N
*Lepidonotothen nudifrons*	B	0.63	2	5.75	0.98/ 2.23/ 1.05 (0.52–2.26)	160.39/ 365.75/ 172.13 (85.26–370.492)	N
*Pagetopsis macropterus*	B	0.49	0.9	2.32	0.45/ 0.93/ 0.49 (0.08–0.94)	48.52/ 99.94/ 52.69 (8.60–101.075)	N
*Trematomus bernacchii*	B	0.48	1.3	4.28	0.67/ 0.49/ 0.78 (0.43–1.29)	83.23/ 60.67/ 96.3 (53.09–159.26)	J
*Trematomus hansoni*	B	0.89	4.2	10.82	1.18/ 1.51/ 1.84 (0.00–57.33)	135.32/ 173.94/ 211.49 (0–6589.66	N
*Trematomus nicolai*	B	0.17	0.5	2.11	0.25/ 0.87/ 4.23 (0.60–4.25)	27.7/ 96.18/ 470 (66.67–472.23)	N
*Trematomus pennellii*	B	0.13	0.3	1.8	0.12/ 0.12/ 3 (0.46–3.50)	10.61/ 10.79/ 263.16 (40.35–307.02)	J
		**S7**					
**Species**	**E**	**hd**	π *10 ^**3**^	ϴW*10 ^**3**^	T(ex) (DSP)/ T(ex) (S)/ T(ex) (A)*10 ^**3**^	**T(ex) (DSP)/ T(ex) (S)/ T(ex) (A) (Kya)**	**DS**
*Aethototaxis mitopteryx*	P	0.77	4.22	14.19	0.67/ 1.92/ 0.88	1117.92/ 3200.67/ 1459.43	N
*Pagothenia borchgrevinki*	P	0.84	3.995	8.5	0.6/ 2.96/ 2.21	1005.12/ 4934.21/ 3691.52	J
*Trematomus newnesi*	P	0.79	7.41	9.62	1.47/ NA/ 10.45	2102.25/ NA/ 14930.72	J
*Notothenia rossii*	I	0.59	6.88	16.66	1.46/ 5.6/ 11.4	2429.08/ 8428.19/ 19002.66	N
*Trematomus eulepidotus*	I	0.99	11.28	23.85	4.27/ 3.94/ 5.57	8530.21/ 7870.09/ 11137.46	N
*Gobionotothen gibberifrons*	B	0.82	17.88	37.4	0.09/ 10.82/ 3.66	121.85/ 15453.78/ 5231.09	N
*Gymnodraco acuticeps*	B	0.85	24.37	32.59	1.17/ 16/ 45.27	1956.65/ 26669.72/ 75454.82	N
*Lepidonotothen nudifrons*	B	0.54	16.79	23.24	0.53/ 8.56/ 36.28	1060.3/ 17115.58/ 72557.79	N
*Pagetopsis macropterus*	B	NA	NA	NA	NA/ NA/ NA	NA/ NA/ NA	N
*Trematomus bernacchii*	B	0.51	2.1	4.28	0.31/ 1.14/ 1.17	515.74/ 1900.11/ 1954.4	J
*Trematomus hansoni*	B	0.91	6.95	15.1	1.74/ 2.77/ 1.22	3486.43/ 5532.36/ 2434.24	N
*Trematomus nicolai*	B	0.67	5.23	8.56	0.42/ 4.33/ NA	692.25/ 7223.48/ NA	N
*Trematomus pennellii*	B	0.52	1.4	1.8	0.47/ 2.8/ 1.28	790.96/ 4661.02/ 2139.83	J

E-eco-group. hd—haplotype diversity. π—nucleotide diversity. W—Watterson’s estimate of theta per site. T(ex) (DSP)/ T(ex) (S)/ T(ex) (A)*103—time of the expansion estimated in DnaSP/ SITES/ Arlequin in mutational time units. T(ex) (DSP)/ T(ex) (S)/ T(ex) (A) (Kya)–absolute time of the expansion estimated in DnaSP/ SITES/ Arlequin in Kya. For time of expansion estimated in Arlequin are confidence intervals in brackets. DS—Data source (N—new in this study. J—Janko et.al. 2007)

Datasets for S7 also displayed negative values of Tajima’s D in all cases and in all but one case for Fu’s F_s_. These values were significantly different from zero in five cases of Fu’s F_s_, and in one case of Tajima’s D (Table 1). Arlequin goodness-of-fit statistics did not reject a sudden expansion model in any dataset. Expansion time estimates were older for the S7 intron than for mitochondrial cyt *b* locus—a pattern observed also in Janko et al. [20] (see Table 2).

Expansion time estimates from geographical regions detected as isolated by AMOVA in *T*. *hansoni* show similar or even younger dating as expansion time estimate obtained from combined datasets. In contrast, the estimate from SITES indicated an old expansion, but the model fitted the data poorly and these estimates are unreliable. For the *Lepidonotothen nudifrons* S7 dataset, estimates of expansion times were also more recent than for the combined dataset.

**Fig 2:**
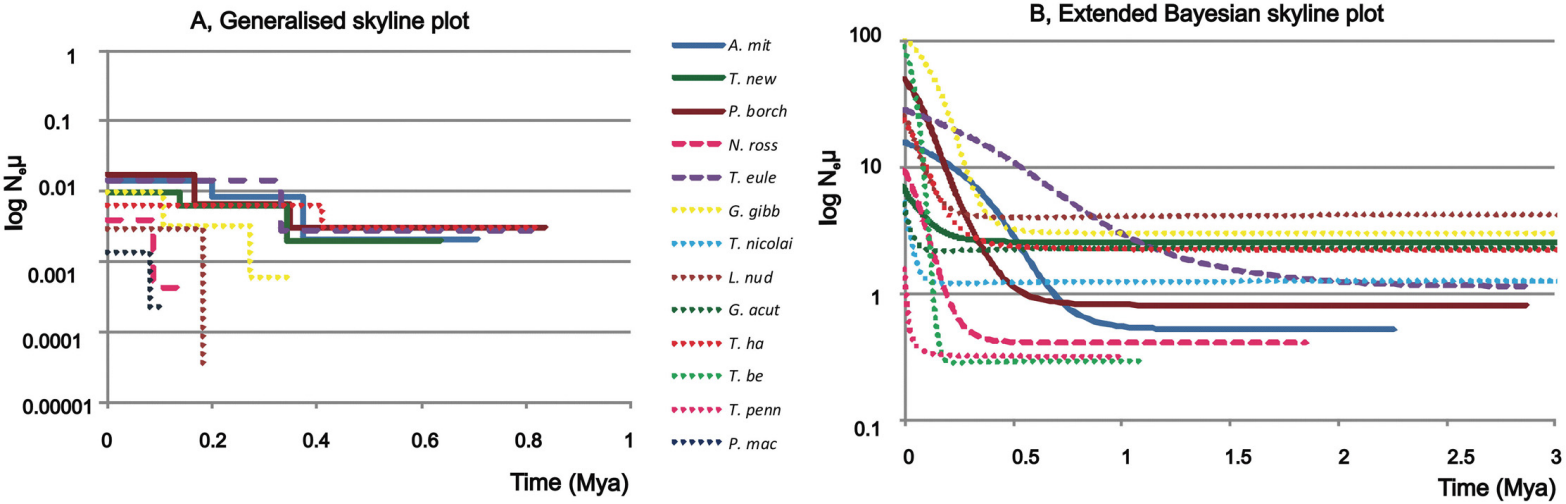
A- Generalised skyline plot of cyt *b* genealogies, B- mean of extended Bayesian skyline plot of combined datasets of cyt *b* and S7 gene. The vertical axis shows the estimated log of N_e_μ and the horizontal axis represents time in Mya; the time is zero now. The pelagic eco-group is indicated by solid lines, intermediate by dashed lines, and benthic by dots (solid lines: blue – *Aethotaxis mitopteryx*, brown – *Pagothenia borchgrevinki*, green – *Trematomus newnesi*, dashed lines: pink – *Notothenia rossii*, violet – *Trematomus eulepidotus*, dots: yellow *Gobionotothen gibberifrons*, dark green – *Gymnodraco acuticeps*, brown – *Lepidonotothen nudifrons*, grey – *Pagetopsis macropterus*, light green – *Trematomus bernacchii*, red – *Trematomus hansoni*, light blue – *Trematomus nicolai*, pink – *Trematomus pennellii*).

Further evidence for past population changes comes from generalized skyline plots. Most cyt *b* datasets showed ε values that grouped adjacent intervals into two or more composite population growth intervals indicating more or less strong population growth (Fig 2A). However, the datasets for *T*. *nicolai*, *T*. *pennellii*, *T*. *bernacchii* and *G*. *acuticeps* contain too little variability for analysis by generalized skyline plots, and produced optimal ε values that grouped all coalescent events into single composite intervals. This prevented any demographic resolution in those species. The generalized skyline plot for the S7 intron showed more coalescence events than for the cyt *b* locus and the resolution extended over longer periods but provided less clear information about the population history (S2 Fig).

Finally, two methods were used to reconstruct demographic history using both loci simultaneously. Lamarc 2.1.10 was used to estimate the parameter g of the growth rate under the model of exponential growth. In all species except *T*. *nicolai*, *G*. *gibberifrons* and *G*. *acuticeps*, 95% support intervals of g excluded zero indicating that populations have been growing (Table 1). In three species (*G*. *gibberifrons*, *G*. *acuticeps and T*. *nicolai*) Lamarc failed to converge even after an extended run (more than 400 000 interactions) and the 95% support intervals for g ranged from negative to positive values. These data should therefore be considered with care, but even in these species, the MLE (maximum likelihood estimate) was always positive (Table 1).

Extended Bayesian skyline plots (EBSP) combining nuclear and mtDNA loci using the branch-specific mutation rates estimated by BEAST indicated that the recent population size of most species increased by at least an order of magnitude from the ancestral population size. (Fig 2B, S3 Fig). However, recent population size estimates of four species (*T*. *newnesi*, *T*. *nicolai*, *T*. *pennellii* and *G*. *acuticeps*) showed only minimal signals of population increase.

### Ecological correlates of demographic differences among species

**Fig 3:**
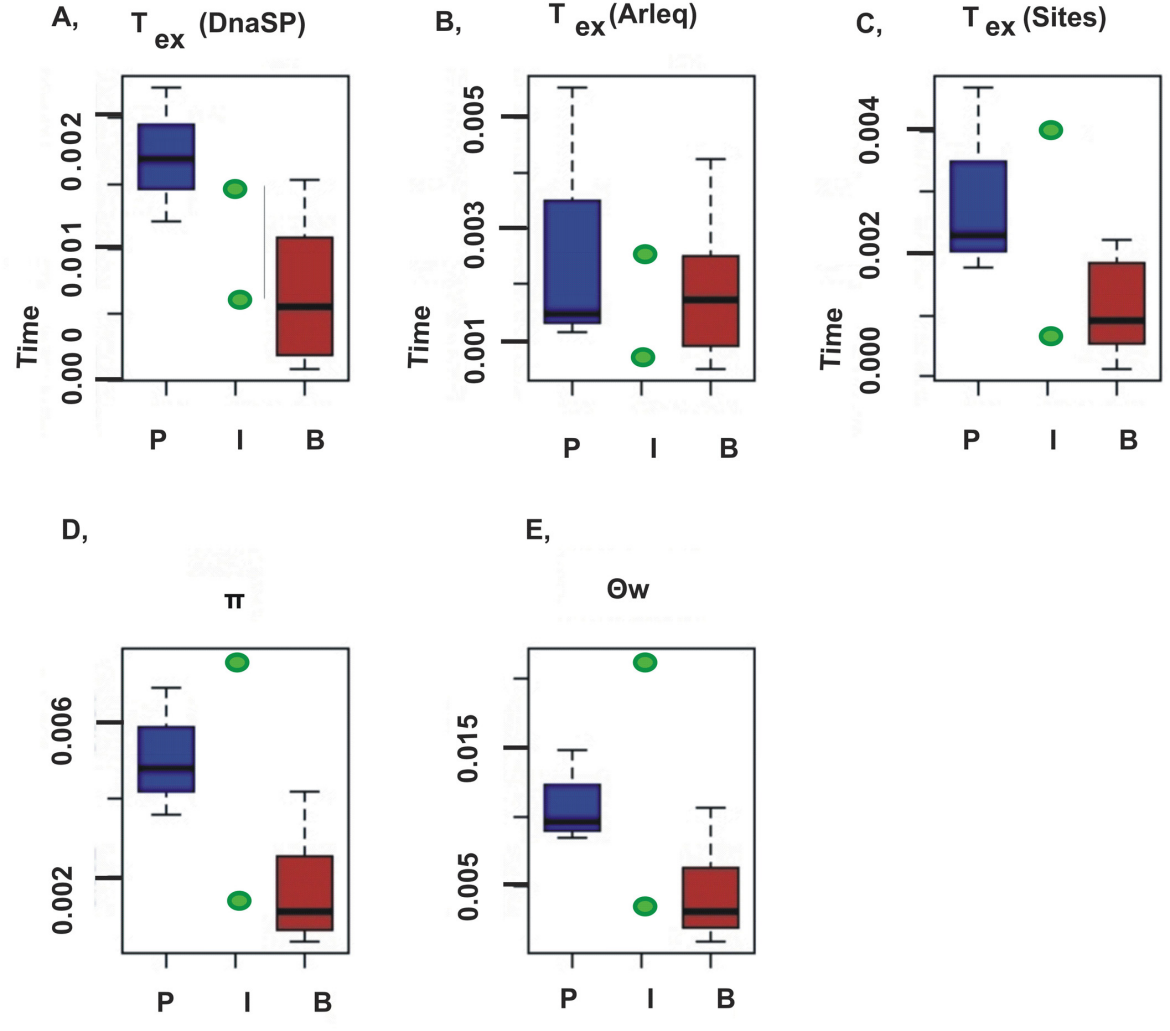
A- C: Comparisons of eco-groups based on cyt *b*: A—T_(ex)_ DnaSP: time of the expansion onset estimated in DnaSP in mutational units, B—T_(ex)_ Arlequin: time of expansion onset estimated in Arlequin in mutational units, C—T_(ex)_ SITES: time of the expansion onset estimated in SITES in mutational units, D—π: nucleotide diversity of cyt *b*, E—θ_W_: Watterson’s estimate of theta per site for cyt *b*. The pelagic group is indicated in blue, intermediate in green and benthic in brown.

Estimates of the genetic diversity (hd, π, θ_W_) and the relative timings of changes of population size varied considerably among species (Table 2, Fig 3). However, unequal sampling does not account for the among-species variability observed since the GLM (generalised linear model) incorporating three factors—sample size, geographical sampling coverage and transect length—did not explain the distribution of the data well and generated higher AIC scores than corresponding null models.

On the other hand, the ecological grouping of the species accounted for a significant portion of the variability in genetic diversity indices and demographic parameters for the cyt *b* dataset. GLMs incorporating eco-group, sample size, sampling region, and geographical transect as a parameter always produced a lower AIC value than the null model (Table 3). Moreover, the ΔAIC was significant in F-tests in ANOVA for the parameter π and the expansion time estimates from DnaSP. The WMW (Wilcoxon-Mann-Whitney test) test suggested significantly lower values in the benthic group for all indices except the estimate from Arlequin.

**Table 3:** The effect of ecological groupings, of sample sizes (n), of sampling region (sample) and of geographical transect (dist) on the distribution of parameter values estimated from cyt *b*.

GLM Formula	AICglm1	AICglm0	ΔAIC	P	WMW
**GLM eco-group**					
Hd	4.736	7.136	-2.4	0.085	0.04848
π	59.134	63	-3.866	0.048	0.02424
θW	83.593	85.78	-2.187	0.092	0.04848
T(ex) (DnaSP)	25.021	29.088	-4.067	0.045	0.02424
T(ex) (Sites)	46.652	48.31	-1.658	0.11	0.04848
T(ex) (Arleq)	52.8	49.7	3.099	0.7	0.7758
**GLM n**					
Hd	8.3	6.33	1.97	0.9	NA
π	-114.7	-116.7	2	0.8	NA
θW	-91.7	-93.56	1.86	0.74	NA
T(ex) (DnaSP)	169.01	167.13	1.88	0.86	NA
T(ex) (Sites)	189	187.2	1.8	0.7	NA
T(ex) (Arleq)	205.75	203.77	1.98	0.9	NA
**GLM sample**					
Hd	8.2	6.33	1.87	0.73	NA
π	-114.8	-116.7	1.9	0.72	NA
θW	-91.6	-93.56	1.96	0.86	NA
T(ex) (DnaSP)	168.6	167.13	1.47	0.5	NA
T(ex) (Sites)	189.16	187.2	1.96	0.65	NA
T(ex) (Arleq)	205.61	203.77	1.84	0.72	NA
**GLM dist**					
Hd	8.1	6.33	1.77	0.69	NA
π	-114.8	-116.7	1.9	0.76	NA
θW	-91.6	-93.56	1.96	0.84	NA
T(ex) (DnaSP)	168.5	167.13	1.37	0.47	NA
T(ex) (Sites)	189.25	187.2	2.05	0.97	NA
T(ex) (Arleq)	205.67	203.77	1.9	0.78	NA

GLM Formula describes the design of each Generalised Linear Model (genetic data were fitted against eco-group, sample size (n), biogeographic provinces (sample) and distance of sampling transect (dist), AICglm1—AIC score for GLM incorporating the eco-group parameter, AICglm0—AIC score of the null model, ΔAIC—difference in AIC values between the two models, P—p value of the ANOVA F test, WMW—provides p-values of Wilcoxon-Mann-Whitney test for differences between pelagic and benthic groups. For the codes of the parameters see Table 2 and for sample sizes see S1 Table.

Population expansion was consistently shown in the EBSP. In particular, for those species where considerable population growth is indicated, we observed a more recent onset of population expansion in benthic species when compared with the pelagic ones. The intermediate group appeared most heterogeneous where *T*. *eulepidotus* showed the oldest signal of population growth (Fig 2B, see S3 Fig for demographic histories with credibility intervals). A similar picture is shown in the generalized skyline plot for cyt *b* (Fig 2A).

## Discussion

The population size of species rarely remains unchanged over long periods. Population genetic studies in Southern Ocean species demonstrate significant deviations from neutral expectations in most of the species studied here. In previous studies this has commonly been attributed to past population fluctuations (reviewed in [5]). Consistent with this expectation, we found significantly negative values of neutrality indices in all cyt *b* datasets and in five of eight S7 datasets (Table 1) coupled with star-shaped haplotype networks (Fig 1) implying population expansion. Lack of significance in values of the HKA and McDonald-Kreitman tests (Table 1) suggest that such patterns were caused by demographic events rather than by locus-specific selection pressure or selective sweeps. Significantly positive g values from multilocus analysis by LAMARC support such a conclusion in all species except *T*. *nicolai* and *G*. *acuticeps* where the confidence intervals included 0. We should also carefully consider the signal in *G*. *gibberifrons* where the chains did not converge after a large number of iterations. More or less recent population growth is also suggested by EBSP (of combined unlinked loci) in all but two species from this study and two from Janko et al. [20] (*T*. *newnesi*, *T*. *pennellii*, *T*. *nicolai*, and *G*. *acuticeps*) (Fig 2B).

Coalescent genealogy samplers like LAMARC or EBSP allow very detailed insights into population histories when datasets contain sufficient variability to parameterize the underlying model or to group adjacent coalescent intervals and to estimate historical population sizes. However, in several cases LAMARC or EBSP did not detect large changes in population size although other demographic analyses (Tajima´s D, Fu´s Fs) indicated likely population expansion in these species. This highlights the value of using a number of different statistical approaches (see also below). It is likely that a number of identical sequences and limited genetic variability resulting in few coalescent events led to the loss of demographic signal [37]. In *T*. *pennellii T*. *nicolai*, and *G*. *acuticeps*, the generalized skyline analysis found only one composite interval, supporting this interpretation. On the other hand, the genetic variability of *T*. *newnesi* was large compared to other species and therefore ESBP plots show that populations of cryopelagic *T*. *newnesi* probably did not dramatically fluctuate in size during the Pleistocene. This is consistent with the finding of Janko et al. [20], who reported a lack of population expansion signals in *T*. *newnesi*.

### Sources of interspecific variability in genetic diversity and demographic patterns

We observed considerable among-species variation in parameters of genetic diversity (hd, π, θ_W_) and demographic history (Table 2, Fig 3). This is typical for studies of Southern Polar fish taxa and for marine species in general [4]. However, in this study we found a clear correlation of lifestyle with among-species variation. This variation is unlikely to result from either unequal geographical sampling or from geographical structuring of these species, either of which can theoretically affect demographic inference. Although AMOVA indicated that some species are structured to a significant level, GLMs incorporating sampling sizes and transect lengths as well as biogeographic coverage, did not account for the distribution of the data. Looking at expansion time estimates of significantly structured fish populations, we did not observe many differences between demographic estimates from sequences originating from particular regions and demographic estimates from total sample sizes. This further suggests that geographical structure does not significantly affect our inferences. On the other hand, partitioning the species according to their life style into the three eco-groups did account for much of the observed variability in population genetic and demographic estimators and greatly improved the model fit to the cyt *b* datasets (Table 3). Pelagic species have significantly higher mtDNA genetic diversity (hd, π) and effective population size (θ_W_) than do benthic ones (parameter values in Table 2, p values in Table 3, Fig 3). The two species with significant AMOVA test values (*T*. *hansoni and L*. *nudifrons*) belong to a benthic group. Population structure within a species tends to increase genetic diversity and inflate the expansion time, but the benthic group still proved significantly less genetically variable and had a more recent expansion time compared to the pelagic group. This supports our view that among-species differences are not caused by geographical structure but instead correlate with ecological clustering.

The higher diversities of pelagic species may be trivially explained by the higher carrying capacity of the pelagic realm than the shallow benthos—therefore supporting higher population sizes of pelagic species. However, given that the estimates of genetic diversity are sensitive to historical population fluctuations, we think that the differences reflect different demographic histories for each eco-group rather than their actual population sizes. Support for this interpretation comes from our finding that a significant portion of the variance in expansion time estimates from cyt *b* (except those from Arlequin) can be attributed to the division of species into the three eco-groups (Table 3). WMW tests also indicated significant differences among population expansion times of pelagic and benthic species. Generalized skyline plots of cyt *b* as well as EBSP analysing both loci simultaneously also show that the onset of demographic expansion of pelagic species generally started earlier than in benthic species (with the exception of *T*. *hansoni* in the cyt *b* dataset and *G*. *gibberifrons*; Fig 2, Table 2). This suggests that benthic and pelagic species tend to consistently differ in their demographic histories.

Three potential problems could complicate the role of ecology on demographic response. First, we observed disagreement between Schneider and Excoffier’s estimator of population expansion time (implemented in Arlequin software [38] and other methods). The moment method of Rogers [39] implemented in DnaSP is sensitive to deviations from the infinite sites model [38] and may underestimate τ in the case of saturated DNA or multiple substitutions occurring at the same site. However, we observed the greatest discordance between τ estimates in species with very low variability, i.e. *T*. *pennellii* (number of segregating sites S = 2), *T*. *nicolai* (S = 3) and *G*. *acuticeps* (S = 6) (see S1 Fig) where such a problem should not occur. Low-variability datasets were also characterized by large confidence intervals around Arlequin τ estimates suggesting that this discrepancy is probably caused by insufficient variability in these datasets.

Second, stable isotope analysis by Rutschmann et al. [34] raised questions about the ecological classification of *T*. *nicolai*. Although there is a possibility that stable isotope analysis does not necessarily provide accurate information about the ecology of the species [40] we repeated the GLM and WMW analysis treating *T*. *nicolai* as a species with an unclear ecological position. We still found significant differences between pelagic and benthic species, as the corresponding GLMs always resulted in lower AIC scores than the null models for all demographic parameters (Hd, π, θ_W_ and estimates of population expansion times).

Finally, when analysing the S7 nuclear locus separately, we found no significant differences between pelagic and benthic species and estimated genetic variability and population expansion times were higher than those from *mt*DNA [39,41], a pattern also seen in other studies [20,42]. The most likely explanation of this finding is that the differences among loci result from the variable properties of each marker. Relatively fast-evolving mitochondrial loci such as cyt *b* [20] are likely to incorporate traces of more recent demographic events than more slowly evolving markers like S7. Moreover, substantial differences in genetic differentiation estimated from nuclear and *mt*DNA markers may arise from different modes of inheritance and ploidy variances [43]. Nuclear markers have longer coalescent times relative to mtDNA due to higher effective population sizes and are therefore expected to preserve demographic history over longer periods. It is therefore possible that deviations from the simple stepwise growth model caused by cyclical population expansions and contractions have a greater effect on the reconstruction of demographic history from the nuclear marker than for the mitochondrial locus. Indeed, Rogers [39] demonstrated that when a population oscillated in size rather than monotonically expanded, the τ estimate of the population growth would mostly reflect the initial growth period during the coalescent history of a given marker. In any case, the combined analysis of both markers (EBSP) indicated a generally more recent onset of expansions in pelagic species.

In a summary, our comprehensive comparative analysis of thirteen fish species provides evidence that lifestyle has a major effect on population genetic structure as well as on the responses to important environmental events such as climate change.

### Role of species ecology in the evolution of the Southern Polar biota

Populations of shallow benthic organisms are likely to have been reduced primarily by physical damage of benthic habitats during glacial maxima. This is likely to have included a combination of lower sea levels, grounded ice, mass wasting, and general ecological factors such as lower food availability, less advection and increased inter-specific competition [28,44]. The response of pelagic species to these processes is less clear. Thatje et al. [25] postulated that Southern Ocean pelagic organisms were probably also restricted to a few areas of local marine productivity (such as polynyas) and that the pelagic realm was somewhat reduced by complete, multi-annual sea-ice coverage resulting in decreased ecosystem productivity. On the other hand, pelagic communities might have profited from the expansion of the pelagic habitat of the Southern Ocean during glacial maxima due to the northward shift of the APF [18]. Smetacek and Nicol [44] also suggested that the apparent reduction of productivity in the seasonal sea-ice zone during cold periods may have been the result of an expansion in the populations of grazers and predators. Our data support these views and suggest that populations of closely related species that differ in their benthic and pelagic specialization had significantly different histories and population expansions most likely driven by different external stimuli.

Applying the fossil-based molecular clock calibration of Near [27] to their data led Janko et al. [20] to propose that major population expansions of the two benthic species coincided with the retreat of the grounded ice, whereas expansion of pelagic populations largely predated the LGM; consistent with our estimated expansion dates (Table 2). However, given the critique by Patarnello et al. [45] we refrain from linking absolute dating of the expansions to presumed climatic events. This is mainly because the application of molecular clocks calibrated with ancient nodes (i.e. older than several Mya as in Near [27] may lead to overestimates in the dating of recent (intraspecific) events [46] making such absolute time estimates biased, especially to recent events. Our absolute time estimates thus probably represent probable upper limits of the true values.

Nonetheless, given that the markers assessed here do not deviate from rate constancy [20], our analysis of closely related species is informative even in relative terms since the degree of overestimation due to the time-dependency of molecular clocks is negatively correlated with the true time of the event [46]. The estimates of relatively young expansion times in benthic species should thus be biased towards even more recent values than those of older events in the pelagic taxa. This suggests that the true differences between the eco-groups may be even greater than detected in our study.

Therefore, our conclusion that much of the among-species variance can be accounted for by differences in lifestyle is valid even without absolute dating. This suggests that reactions to important extrinsic events such as climate change, depend on species-specific traits. This conforms to the hypothesis that benthic populations were reduced by negative effects of glaciations and recovered recently after these negative effects vanished. On the other hand, pelagic species probably reacted differently as shown by more ancient estimates of population expansions.

Combining current population demographic data with previously published macro-evolutionary patterns allows deeper insights into how Southern Oceanic species might have survived the Pleistocene. Although such climatic events probably increased the risk of local population extinctions—as suggested by signals of population expansion from low effective population sizes [29,42,47]–the overall net diversification rate was probably not altered. A multilocus phylogenetic analysis by Janko *et al*. [26] found no evidence for either an increase or decrease in the diversification rate of the Trematominae during the Pleistocene. This indicates that although local populations probably went extinct or were drastically reduced in size during unfavourable periods, the extinction rates of entire species overall may not have changed.

Thatje et al. [28] provided an explanation as to how species might have survived Antarctic glaciations. They suggested that asynchronous patterns of ice expansion and retreat in different Antarctic regions during glacial periods allowed the survival of benthic species by migration between temporarily available refugia along the continental shelf. Although Van de Putte et al. [48] found greater geographical structure in benthic Trematominae species compared to pelagic *T*. *newnesi*, and Damerau et al. [12] reported restricted gene flow among populations of *Lepidonotothen larseni*, the population structure of notothenioid species is generally weak [12,14,36,48–51]. This supports the view that these species had a sufficiently high dispersal rate to allow effective migration among temporarily available refugia along the continental shelf. Combined microevolutionary (this study) and macroevolutionary [26] analysis of Trematominae is therefore in line with Thatje et al.’s [28] hypothesis and suggests that species that survived inhospitable periods and glacier-induced extinctions may have experienced significant local effects.

Overall, our data support the hypothesis that benthic species contain both lower genetic diversity and were subject to more recent major population expansions than pelagic species. This may reflect population reductions during glacial periods followed by population expansions during climate warming. These findings indicate that the ecological specialization of a species may have an important effect on its response to climatic shifts.

## Materials and Methods

### Sampling

Approval to catch fish to take fin clips and to return the fish alive was granted by the Animal Ethics Committee of the University of Otago AEC 56/2002 and the Ministry of Foreign Affairs and Trade who issued permits under the Antarctic Marine Living Resources Act. Collecting samples was also permitted by the Ministry of Environment of the Czech Republic, as the competent governmental authority pursuant to Sections 8 and 24 of Act No. 276/2003 Coll.

**Fig 4:**
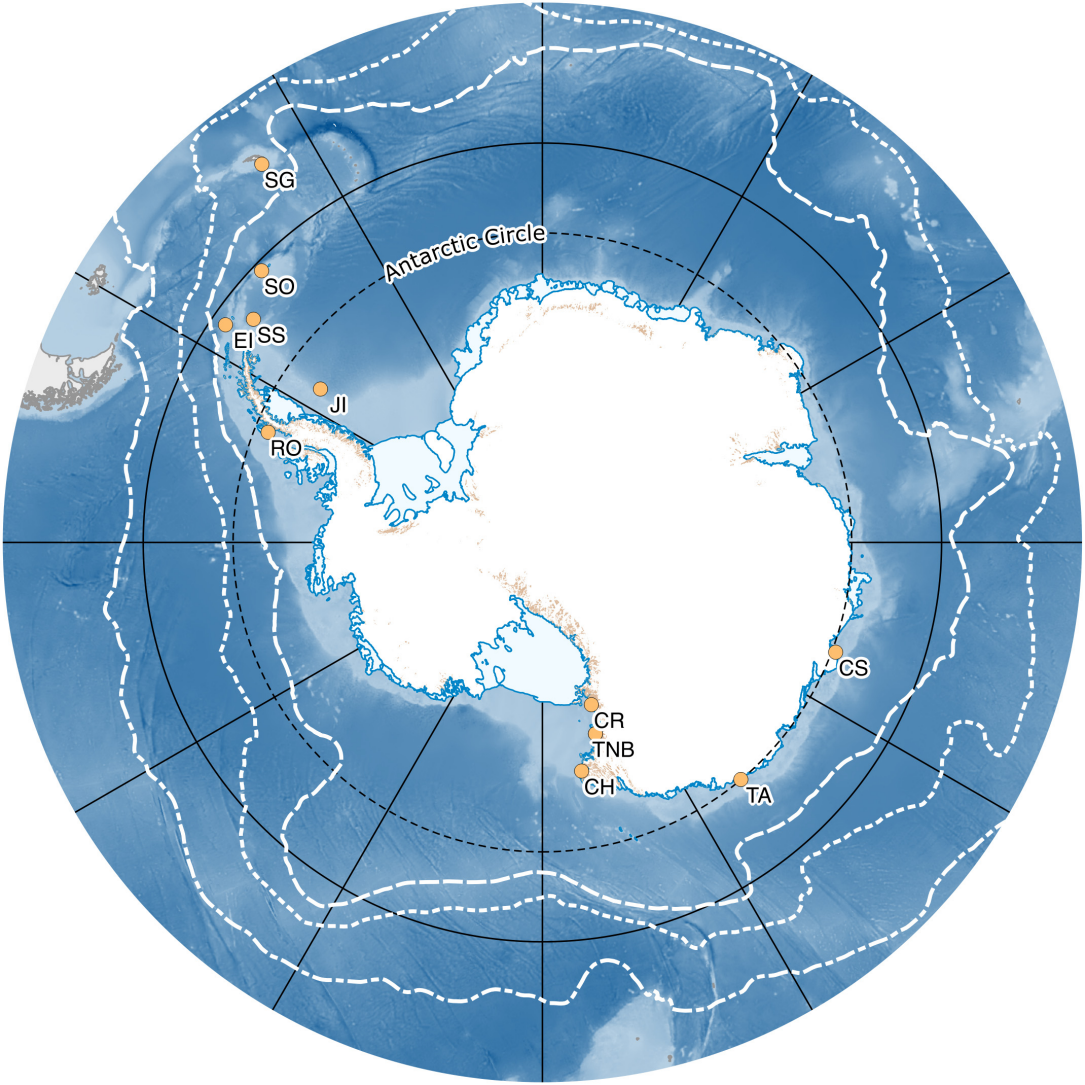
Map of Antarctica showing sampling locations. SO—South Orkney, SG—South Georgia, EI—Elephant Island, SS—South Shetlands, JI—Joinville Island, CR—Cape Roberts, CH—Cape Hallett, TA—Terre Adelie, TNB—Terra Nova Bay, Rothera—Rothera research station, CS—Casey, CA—Cape Armitage.

Samples of shelf notothenioid fish were collected by bottom trawl or fishing rod from eight Antarctic locations including the Antarctic Peninsula, maritime Antarctic and Ross Sea area (see Fig 4 and S1 Table) during the cruise ANT-XXIII/8 with RV Polarstern in 2006, and during expeditions supported by Antarctica New Zealand (event K066), and the Ukrainian Antarctic programs. The data for four species from Janko et al. [20] were added to the analyses described here (two cryopelagic species: *Pagothenia borchgrevinki*, *Trematomus newnesi*, and two benthic species: *Trematomus pennellii* and *Trematomus bernacchii*).

Species were allocated into one of three ecological groups (eco-groups) according to their lifestyle (see S2 Table) determined by such characteristics as adult foraging behaviour, spawning strategy, egg type, and larval and juvenile dependence on the marine bottom (substrate). Guarding and incubation time of benthic eggs was judged as a key trait for the benthic eco-group classification [52] because it predetermines the vital dependence of the species on available benthic habitat.

The pelagic/cryopelagic (P) eco-group comprises species foraging in the water column and with pelagic eggs (*Aethotaxis mitopteryx*, *Pagothenia borchgrevinki*, *Trematomus newnesi)*.

The benthic eco-group (B) comprises species foraging in the benthos and with benthic eggs (*Trematomus hansoni*, *Trematomus nicolai*, *Gobionotothen gibberifrons)* and species known to guard nests (*Lepidonotothen nudifrons*, *Gymnodraco acuticeps*, *Pagetopsis macropterus*, *Trematomus pennellii*, *Trematomus bernacchii*).

Finally, the intermediate (I) eco-group was developed for species where the classification is either ambiguous or unknown and so were classified separately. This group includes *Trematomus eulepidotus*, which is known to forage in the water column but is reproductively bound to the bottom as it lays eggs in sponges, and *Notothenia rossii*, where there is a switch from pelagic eggs to benthic juveniles and semipelagic adults (see S2 Table).

The classification of *Trematomus nicolai* is not without ambiguity, since stable isotope analysis by Rutschmann [34] suggested that it is pelagic. However, given the limitations of the isotope-based inference [40] we have used the traditional benthic classification of *Trematomus nicolai* [53] (see S3 Table), further supported by its low buoyancy comparable to other clearly benthic fish [54].

### Molecular markers and techniques

Two loci were PCR amplified; the mtDNA gene for the cyt *b*, and the first intron in the nuclear S7 gene. Cyt *b* was amplified according to two species-specific amplification protocols and primer combinations (see S2 Table) that gives an approximately 600 bp fragment. Approximately 700 bp of the S7 intron were amplified according to the protocol of Janko et al. [20]. Homologous sequences were aligned using CLUSTALX software and any ambiguities resolved by inspection of the sequencing chromatograms. Sequences were deposited in the GenBank database, accession numbers: cyt *b*, KC153538—KC153654; S7 intron, KC520782- KC520827, KC833756—KC833898. The S7 intron sequences of *Notothenia rossii* and *Lepidonotothen nudifrons* were not long enough to be deposited in the Genbank, as the length of the longest fragment without recombination was below the minimum length required by NCBI (>200 bp) (enclosed as S3 Table).

### Phase resolution

Recombination may complicate both the resolution of gametic phases and the inference of demography. To avoid this problem, we initially aligned only S7 sequences from homozygous individuals within each species-specific dataset and applied the “four-gamete test” [55] implemented in DnaSP v. 5.0 [56] to detect potential recombination events. When intralocus recombination was detected, we discarded the sites left or right of the putative recombination events as appropriate in order to retain the longest possible contiguous unrecombined sequence. We subsequently used a Bayesian statistical method [57] provided by fastPHASE implemented in DnaSP to reconstruct the gametic phases of heterozygous individuals in the unrecombined alignment. For a detailed explanation see Janko et al. [26].

### Detection of selection and potential geographic structure

To test whether the variability of each locus was significantly affected by selection rather than by demographic processes, we performed two tests. The McDonald-Kreitman test [58] assesses whether the ratio of silent to replacement polymorphisms in the cyt *b* is the same as the ratio of silent to replacement fixed differences. The significance of departures from neutral expectations was evaluated by the ‘G test of independence’ implemented in DnaSP. We also used the HKA test [59] using both cyt *b* and S7 data for each species. This test compares the observed number of segregating polymorphisms within species to the number of differences between species. In a neutral case, the number of intraspecific polymorphisms and interspecific divergence are correlated in all loci. The significance of the sums of the deviations was evaluated against a neutral distribution generated from 1000 coalescent simulations with the software HKA. In all cases, *Eleginops maclovinus*, a basal species to all AFGP-containing notothenioids [27] was used as an outgroup. The significance of the outcome (0.05) was evaluated after sequential Bonferroni correction for multiple testing [60].

We used Analysis of Molecular Variance (AMOVA) [61] implemented in Arlequin to test for potential geographic subdivision of examined species using the best-fitting model of sequence evolution for each species. We estimated the level of genetic structuration within populations, between populations within a region (in all species sampled at at least two sites) and between regions (wherever sampling was from separate regions). We defined the regions according to biogeographic provinces defined by Barnes et al. [62].

### Genetic diversity and inferences of past population size change

Each alignment was characterized by haplotype diversity (hd), mean nucleotide divergence (π) and Watterson’s estimate of effective population size θ_W_ estimated with the program DnaSP. In order to robustly evaluate possible differences among eco-groups, we adopted several approaches to test whether the populations of studied species remained constant in size or fluctuated over their demographic history. For all species, minimum spanning networks were reconstructed using the algorithm of statistical parsimony implemented in the TCS program, version 1.06 [63]. Recent population expansions were expected to show star-like phylogenetic patterns in this analysis.

Neutrality tests are often used to test population stability due to their sensitivity to past population size changes that may cause deviations from the mutation-drift equilibrium. Tajima’s D [64] and Fu’s F_S_ test [65] are considered to be two of the strongest tools in detecting traces of past population expansion [56] and were performed in DnaSP with 1000 coalescent simulations to evaluate whether the observed values might result from a population in mutation-drift equilibrium. The significance of p-values was evaluated by sequential Bonferroni corrections for multiple testing [60].

Estimates of times of the major demographic events were obtained by fitting models of sudden population expansion using two methods. First, we used mismatch distribution for the timing of expansion using the parameter τ [39] with a stepwise population growth model. The relationship between absolute time (t) and τ is: *t* = τ/2μ (where μ equals the substitution rate per locus, rather than per site). Values of τ were estimated by two programs; DnaSP and Arlequin version 3.11 [66]. The latter program also allows an estimate of τ [38] with confidence intervals obtained by a parametric bootstrap approach based on 1000 replicates.

Second, the maximum likelihood method implemented in SITES [67] takes into account the distribution of polymorphisms in a population and estimates three parameters of a model of sudden population expansion, these are θ_ancestral_, θ_final_ and τ.

Due to known problems in dating of recent events in general [46] and in the application of molecular clocks to notothenioids in particular [27], we have used mutational time units to compare expansion times estimates among species (see Discussion). Since expansion times provided by mismatch analyses and SITES are scaled by per-locus mutation rates, rather than per site, we divided our τ values by the locus sequence length in each species to obtain comparable values. We also used the estimates of branch-specific mutation rates inferred with BEAST (see below how BEAST was used) to translate the mutation time estimates into absolute dating.

Likelihood Analysis with Metropolis Algorithm using Random Coalescence, LAMARC *2*.*1*.*10* [68] was used to estimate effective population exponential growth rate. To reduce a bias of growth rate (g) both genes were analysed simultaneously. The relative effective population size was set to 1 for mitochondrial gene and to 4 for nuclear. These settings allowed us to estimate Θ on the *mt*DNA scale. The relative mutational rate used was four times higher for cyt *b* than for S7 gene.

LAMARC analyses were performed more than once with a different random number seed each time to assess whether 95% of the ML point estimates fell within each other's confidence intervals. LAMARC was run for at least 400 000 iterations, and longer in datasets where convergence was difficult to reach.

As the final approach, the demographic history of the sampled DNA sequences was investigated through two versions of skyline plots; the generalized skyline plot and the extended Bayesian skyline plot (EBSP). The generalised skyline plot [37] analyses each locus separately and is helpful in testing whether the variability of datasets is sufficient for reconstruction of more than one coalescence interval. We generated ultrametric genealogies for each species in PAUP* 4.0 [66] under the strict clock model and used Genie v3.0 [69] for skyline plot reconstruction. The optimal value of the parameter ε, which controls the grouping of adjacent coalescence intervals, was chosen by maximizing the log L_AICc_ function of the skyline plot. Since each locus was analysed separately for each species, the population size estimates of the generalized skyline plots are scaled in mutation time units.

The EBSP [70] implemented in BEAST v. 1.8.0. [71] enables the combination of multiple loci for population dynamics assessment, which can reduce the variability in estimating the population size function. The combination of both loci requires an estimate of relative locus-specific substitution rates in each species. To assess the substitution rate estimates of each marker, a reduced dataset of cyt *b* and S7 was produced. Datasets of both markers, cyt *b* and S7, comprised one sequence chosen at random from each of the thirteen studied species and one sequence of the basal Trematominae species *Trematomus scotti* [26]. The divergence time of *T*. *scotti* from the remaining Trematominae was used according to the timing of Near [27]. The sequence evolution model was set to HKY (estimated by jModelTest v 2.1.2 [72] based on the Akaike information criterion (AIC)). Given that Janko et al. [26] found no evidence for significant background extinction rate in Trematominae, a Yule process of speciation was used with an uncorrelated lognormal relaxed clock. The normally-distributed priors for uncorrelated lognormal relaxed clock mean substitution rate were applied: 0.98% for cyt *b* and 0.28% for S7 gene per site per Mya with standard deviation of 0.5 for cyt *b* and 1 for S7 marker. The standard deviation of the mean was left at the default. Four independent MCMC runs for each locus and species were performed with the length set to 10^7^ and MCMC chain convergence was assessed by Tracer 1.5 [73]. Runs were subsequently combined with LogCombiner using 10% burnin, and substitution rate of the terminal branch leading to a species was recorded for each of thirteen species for both markers.

Branch-specific median substitution rates and corresponding 95% HPD intervals obtained with BEAST were used as clock rate priors with normal distribution in the subsequent EBSP analyses combining both loci in each species. Substitution rates for the nuclear locus were scaled relative to the mtDNA rate. A strict clock [74] and piecewise linear model was applied. The best-fitting substitution model was estimated using AIC in jMmodelTest v2.1.2. [72]. The length of the MCMC chain was set to 5×10^8^ and convergence was assessed using Tracer 1.5. Due to the combination of two loci with different mutation rates, the EBSP were scaled in years as provided by BEAST rather than in mutation time units.

### The effects of ecological specialization and geographical sampling on estimates of genetic diversity

To evaluate whether differences in genetic diversity and demographic patterns among species are caused by unequal sampling bias or by differences among fish eco-groups, we characterized each species by four traits: a) number of samples per species, b) length of sample transect measured as a coastline distance with Google Earth [75] c) the biogeographic provinces covered by the sampling (provinces were defined according to more detailed biogeographic segmentation [62]: Eastern Antarctica, Antarctic Peninsula and the Scotia Arc) and d) the eco-group.

Generalized linear models (GLM) with Gaussian error distribution were used to detect traits explaining among-species variability in genetic indices and demographic patterns. We performed GLM forward selection starting from the null model expecting no dependence of measured values on species-specific traits. To do so, the best model was considered to be that with the lowest AIC score and we evaluated its significance by ANOVA using the F test. We also used a non-parametric Wilcoxon-Mann-Whitney test (WMW) to test the differences between the groups of clearly benthic and pelagic species (i.e. not considering the intermediate group).

## Supporting Information

S1 Fig Analyses showing the discrepancies among time of expansion in mutational units estimated from different demographic methods from cyt *b*. The Y-axis shows the logarithm of the ratio of expansion time estimates (in mutational time units) obtained with different methods (DSP—DnaSP, S—SITES, A—Arlequin); X-axis indicates the number of segregating sites within the dataset. The species with highest discrepancies between Arlequin and other estimates are denoted by coloured points: red–*Trematomus pennellii*, green–*Trematomus nicolai*, blue–*Gymnodraco acuticeps*.
(TIF)

S2 Fig Skyline plot of S7 intron genealogies of the pelagic, intermediate, benthic eco-group. The vertical axis shows estimated N_e_μ and the horizontal axis represents time in mutational time units; time is zero in the present. Low variability at this locus resulted in resolution of just one composite interval for several species (*T*. *bernacchii*, *T*. *pennellii*, *G*. *acuticeps*, *L*. *nudifrons* and *P*. *macropterus*), thus preventing their demographic reconstruction.
(PDF)

S3 Fig Demographic changes of studied species estimated by EBSP showing changes in effective population size (Neμ) and credibility intervals over time (measured in Mya). The Y- axis shows estimated effective population size (Neμ), the X-axis represents time in Mya, time is zero at the present. *Pagetopsis macropterus* is not included in the analysis because of the high number of recombination sites in the S7 dataset.
(PDF)

S1 File **Table A:** AMOVA analysis of all newly sequenced species with specimens from more than one locality (*Aethotaxis mitopteryx* was sampled just at EI) (supplement of Janko et al. (2007)); *Trematomus hansoni* geographic subdivision was used according to the structure found by Van de Putte et al. (2012) and locality JI with two samples for *Lepidonotothen nudifrons* was omitted. **Table B:** neutrality tests for species with identified geographical structure separately for both markers. E—eco-group (P—pelagic, I—intermediate, B—benthic). P value—P value of certain fixation indices. Locality: SO—South Orkney, SG—South Georgia, EI—Elephant Island, SS—South Shetlands, JI—Joinville Island, CR—Cape Roberts, CH—Cape Hallett, TA—Terre Adelie, Rothera—Rothera research station, CS—Casey. N—number of haploplotypes. L—length in base pairs of the non-recombined locus. hd—haplotype diversity. π –nucleotide diversity. ϴW—Watterson’s estimate of Theta per site. T(ex) (DSP)/ T(ex) (S)/ T(ex) (A)*10^3^ –time of the expansion estimated in DnaSP/ SITES/Arlequin in mutational time units. T(ex) (DSP)/T(ex) (S)/T(ex) (A) (Kya)–absolute time of the expansion estimated in DnaSP/SITES/Arlequin in Kya. NS—not significantly different from zero. #–values significant at α = 0.05 after sequential Bonferroni correction. * model fits poorly.
(XLSX)

S1 Table Specimen numbers for cyt *b*/ S7 sampled at different locations. (see Fig 4 captions for location details).
(XLSX)

S2 Table References to eco-groups characteristics and primers used in this work. The amplification protocols and primers used for cyt *b* are indicated by references: [1], [2], Reference to E—references to eco-groups integration. The S7 intron was amplified using primers S7RPEX1F and S7RPEX2R [3].
(DOCX)

S3 Table *Notothenia rossii* and *Lepidonotothen nudifrons* haplotypes of S7 intron without recombination events. (TXT)

## Abbreviations

APF: Antarctic Polar Front
LGM: Last Glacial Maximum
AFGP: antifreeze glycoproteins
cyt b: cytochrome *b*
HKA test: Hudson–Kreitman–Aguade´ Test
EBSP: Extended Bayesian skyline plot
BEAST: Bayesian Evolutionary Analysis Sampling Trees
HKY: Hasegawa, Kishino and Yano DNA substitusion model
AIC: Akaike information criterion
MCMC: Markov chain Monte Carlo method
GLM: Generalized linear models
ANOVA: Analysis of variance
WMW: Wilcoxon-Mann-Whitney test
