# Supplementary figures and images for: Lifestyle and Ice: The Relationship between Ecological Specialization and Response to Pleistocene Climate Change
Source: PLoS One. 2015 Nov 4;10(11):e0138766. doi: 10.1371/journal.pone.0138766 (PMC4636791; doi:10.1371/journal.pone.0138766)

A, Pelagic

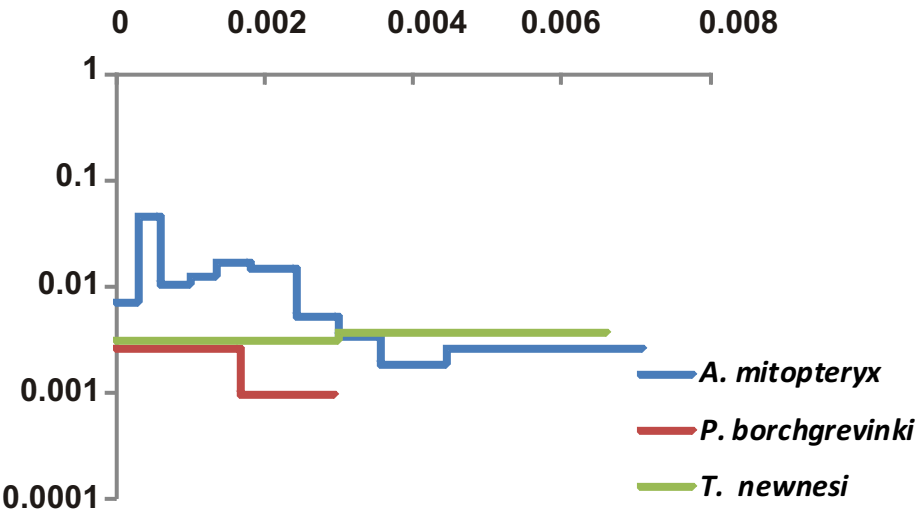

B, Intermediate

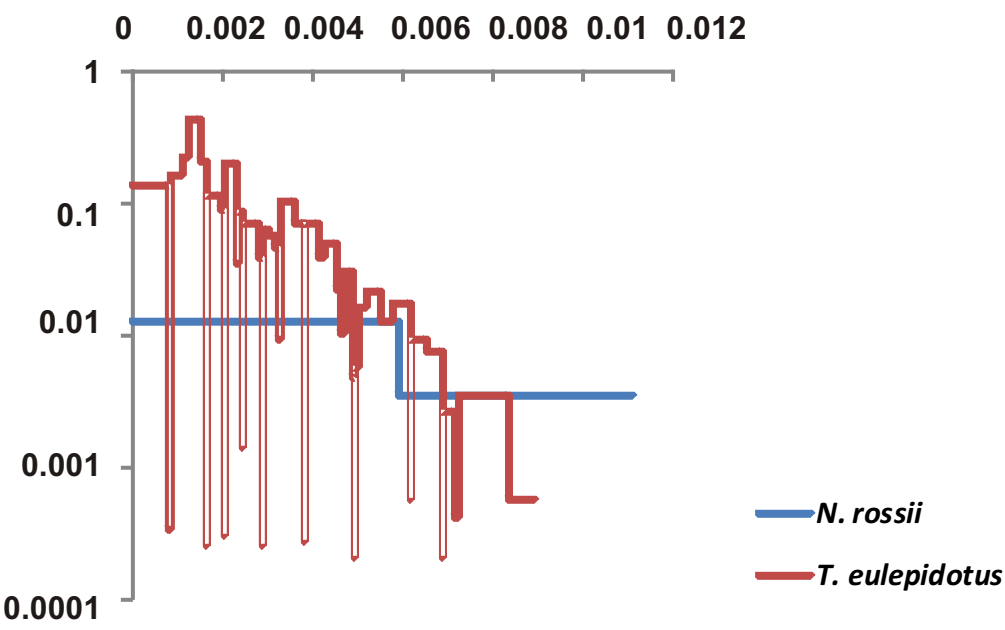

C, Benthic

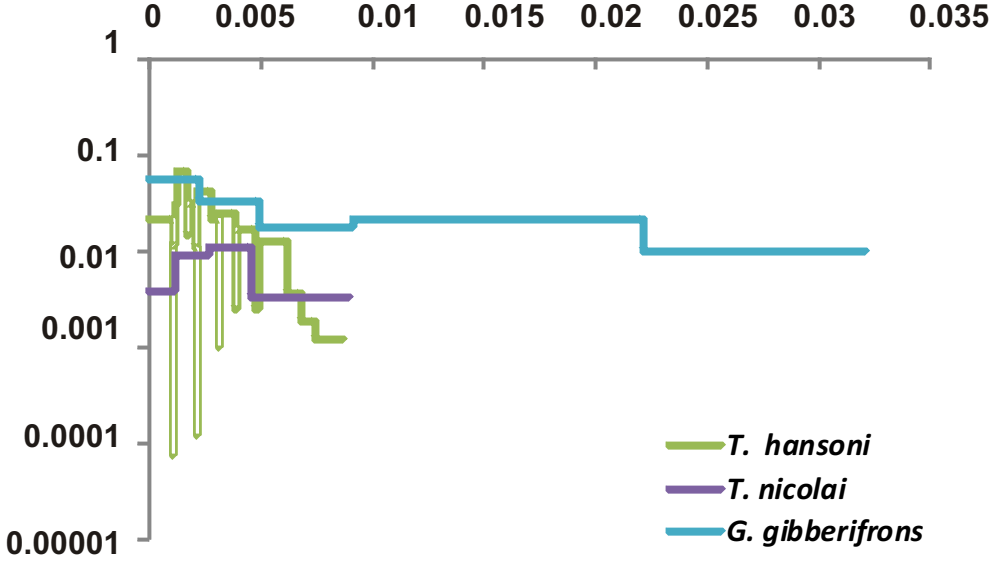

Supplement: S2 Fig — The vertical axis shows estimated Neμ and the horizontal axis represents time in mutational time units; time is zero in the present. Low variability at this locus resulted in resolution of just one composite interval for several species (T. bernacchii, T. pennellii, G. acuticeps, L. nudifrons and P. macropterus), thus preventing their demographic reconstruction. (PDF) [file pone.0138766.s002.pdf]

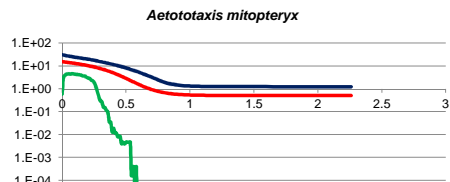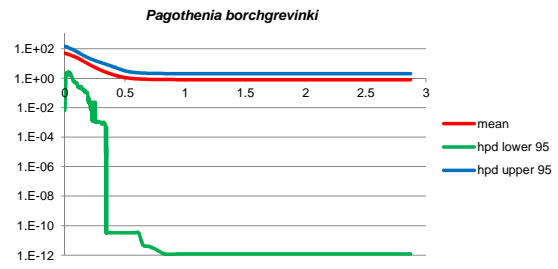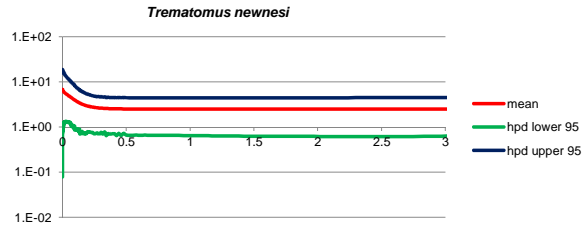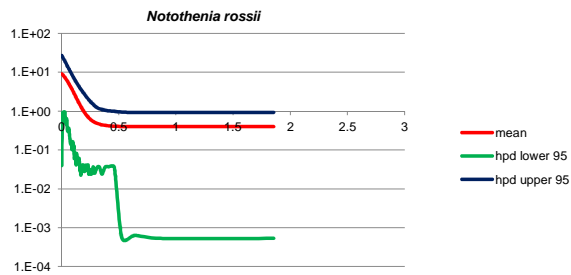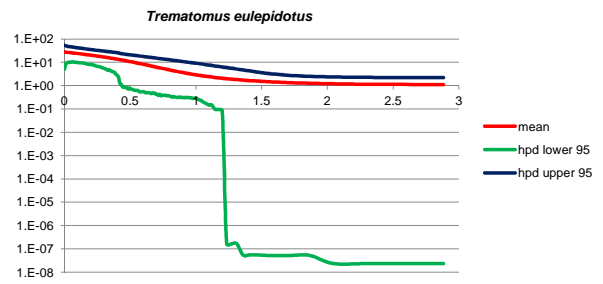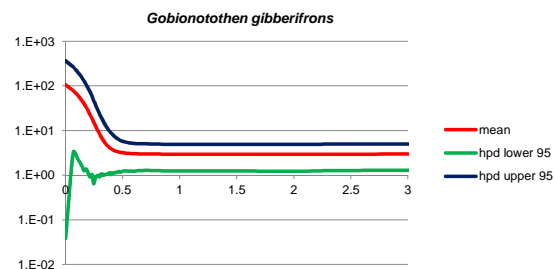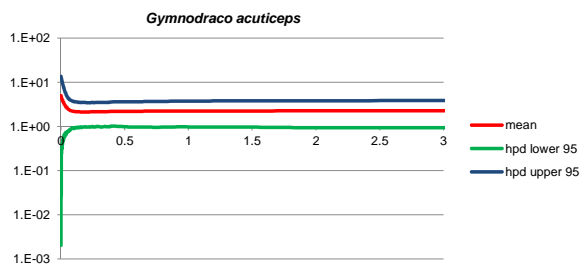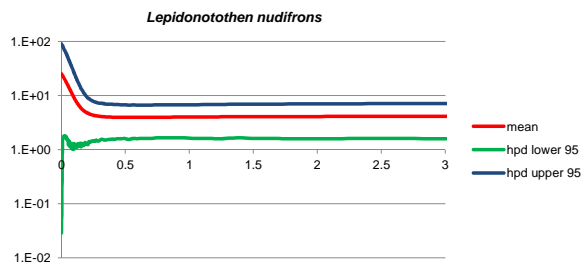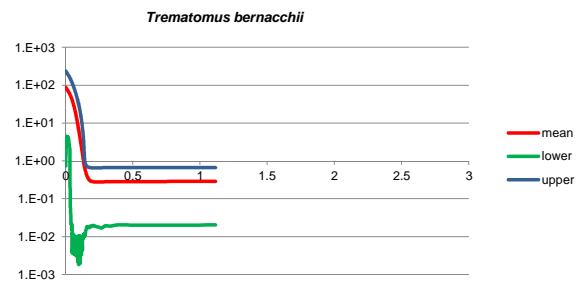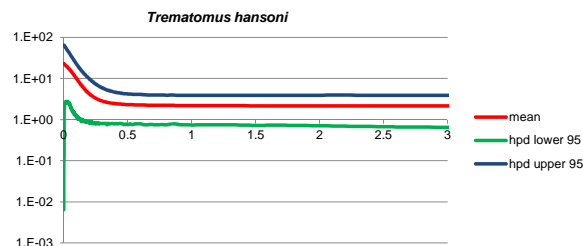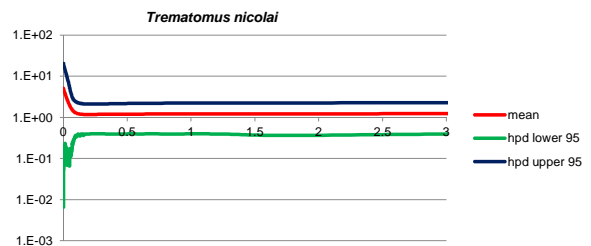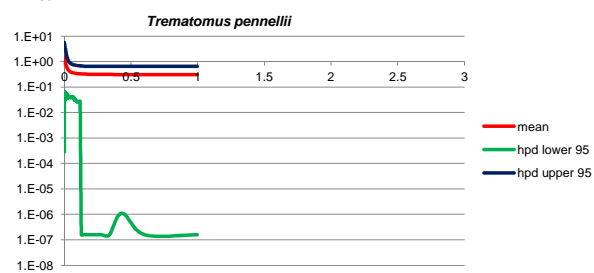

Supplement: S3 Fig — The Y- axis shows estimated effective population size (Neμ), the X-axis represents time in Mya, time is zero at the present. Pagetopsis macropterus is not included in the analysis because of the high number of recombination sites in the S7 dataset. (PDF) [file pone.0138766.s003.pdf]
