# Supplementary material for: Lifestyle and Ice: The Relationship between Ecological Specialization and Response to Pleistocene Climate Change
Source: PLoS One. 2015 Nov 4;10(11):e0138766. doi: 10.1371/journal.pone.0138766 (PMC4636791; doi:10.1371/journal.pone.0138766)
Supplement: S2 Table — The amplification protocols and primers used for cyt b are indicated by references: [1], [2], Reference to E—references to eco-groups integration. The S7 intron was amplified using primers S7RPEX1F and S7RPEX2R [3]. (DOCX) [file pone.0138766.s006.docx]

| **S2 Table: References to eco-groups characteristics and primers used in this work**. Used amplification protocols and primers for cyt *b* are indicated by references: [1], [2], Reference to E – references to eco-groups integration. The S7 intron was amplified using primers S7RPEX1F and S7RPEX2R [3] | | | | |  |
| --- | --- | --- | --- | --- | --- |
| **Species** | **Primers** | **Eco-group** | **Description** | **Reference to E** |  |
| *Aethotaxis mitopteryx* | [1]L 15926; H 15930 | P | Pelagic/cryopelagic: species foraging in the water column and with pelagic eggs | [4],[5],[6],[7] |  |
| *Pagothenia borchgrevinki* | [2]L 16317, U 15786 |  |  | [4], [8], [5] |  |
| *Trematomus newnesi* | [2]L 16317, U 15786 |  |  | [4], [5], [7] |  |
| *Notothenia rossii* | [1]L 15926, H 15930 | I | Intermediate group: species with ontogenetically variable life cycle | [4], [9], [5], [7] |  |
| *Trematomus eulepidotus* | [2]L 16317, U 15786 |  |  | [4], [9], [8], [5], [7] |  |
| *Gobionotothen gibberifrons* | [1]L 15926, H 15930 | B | Benthic species: foraging predominantly in the benthos and often known to actively guard their benthic nests | [4], [10], [5], [7] |  |
| *Gymnodraco acuticeps* | [1]L 15053, H 15930 |  |  | [4], [10], [5] [7] |  |
| *Lepidonotothen nudifrons* | [2]L 16317, U 15786 |  |  | [4], [10], [5] [7] |  |
| *Pagetopsis macropterus* | [1]L 15926, H 15930 |  |  | [4], [10], [5] |  |
| *Trematomus bernacchii* | [2]L 16317, U 15786 |  |  | [4], [8], [5] |  |
| *Trematomus hansoni* | [2]L 16317, U 15786 |  |  | [4], [5], [11], [7] |  |
| *Trematomus nicolai* | [2]L 16317, U 15786 |  |  | [4], [12],[13] |  |
| *Trematomus pennellii* | [2]L 16317, U 15786 |  |  | [4],[5] |  |
|  |  |  |  |  |  |
| **References to Supporting Information Table S2:** | |  |  |  |  |

1. Derome N, Chen W-J, Dettaï A, Bonillo C, Lecointre G (2002) Phylogeny of Antarctic dragonfishes (Bathydraconidae, Notothenioidei, Teleostei) and related families based on their anatomy and two mitochondrial genes. Mol Phylogenet Evol 24: 139–152.

2. Sanchez S, Dettaï A, Bonillo C, Ozouf-Costaz C, Detrich HW, et al. (2006) Molecular and morphological phylogenies of the Antarctic teleostean family Nototheniidae, with emphasis on the Trematominae. Polar Biology 30: 155–166.

3. Chow S, Hazama K (1998) Universal PCR primers for S 7 ribosomal protein gene introns in fish. Molecular Ecology 7: 1255–1256.

4. Eastman JT (1993) Antarctic fish biology: evolution in a unique environment. Academic Press, Inc.

5. Kock K-H (1992) Antarctic Fish and Fisheries. Cambridge University Press. 375 p.

6. Kunzmann, Zimmermann C (1992) Aethotaxis mitopteryx, a high-Antarctic fish with benthopelagic mode of life. Marine Ecology Progress Series 88: 33–40.

7. Rutschmann S, Matschiner M, Damerau M, Muschick M, Lehmann MF, et al. (2011) Parallel ecological diversification in Antarctic notothenioid fishes as evidence for adaptive radiation. Mol Ecol 20: 4707–4721.

8. Klingenberg CP, Ekau W (1996) A combined morphometric and phylogenetic analysis of an ecomorphological trend: pelagization in Antarctic fishes (Perciformes: Nototheniidae). Biological Journal of the Linnean Society 59: 143–177.

9. Gon, O., P. Heemstra (1990) Fishes of the Southern Ocean. J L B Smith Institute of Ichthyology, Grahamstown.

10. Kock K, Pshenichnov L, Devries A (2006) Evidence for egg brooding and parental care in icefish and other notothenioids in the Southern Ocean. Antarct Sci 18: 223–227.

11. Near TJ, Jones CD, Eastman JT (2009) Geographic intraspecific variation in buoyancy within Antarctic notothenioid fishes. Antarctic Science 21: 123–129.

12. Eastman J, Sidell B (2002) Measurements of buoyancy for some Antarctic notothenioid fishes from the South Shetland Islands RID A-9786-2008. Polar Biol 25: 753–760.

13. Kuhn KL, Near TJ, Jones CD, Eastman JT (2009) Aspects of the Biology and Population Genetics of the Antarctic Nototheniid Fish Trematomus nicolai. Copeia: 320–327.
